# SARS-CoV-2 Variants of Concern Delta: a great challenge to prevention and control of COVID-19

**DOI:** 10.1038/s41392-021-00767-1

**Published:** 2021-09-27

**Authors:** Maochen Li, Fuxing Lou, Huahao Fan

**Affiliations:** College of Life Science and Technology, Beijing University of Chemical Technology, Beijing, China

**Keywords:** Vaccines, Infectious diseases, Infectious diseases, Vaccines

Recently, three groups evaluated the effects of monoclonal antibodies, convalescent serum, and vaccines on Delta variant,^1–3^ which leads to concerns about the effectiveness of current vaccines to the upcoming SARS-CoV-2 variants. The World Health Organization (WHO) has designated SARS-CoV-2 variants Alpha(B.1.1.7), Beta(B.1.351), Gamma(P.1), and Delta(B.1.617.2) as Variants of Concern (VOC), among which Delta variant with remarkable transmission and immune escape ability has attracted great attentions.

**Fig. 1:**
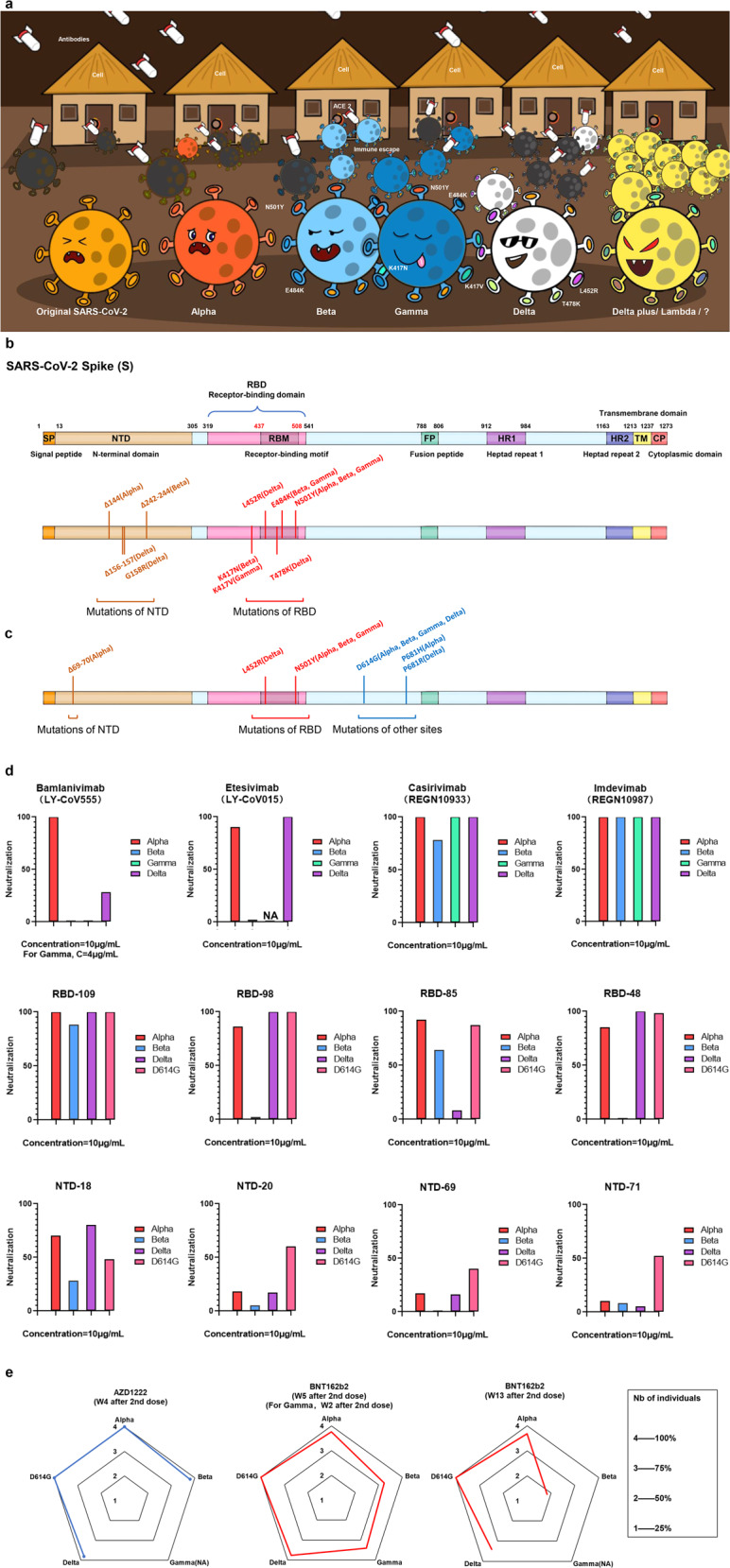
A schematic illustration of the characteristics of VOCs and the neutralization of approved antibodies and vaccine sera. **a** WHO has designated Alpha, Beta, Gamma, and Delta variants as Variants of Concern (VOC), and they are colored in red, light blue, dark blue, and white, respectively. Orange represents the original strain and yellow represents the super-variant (Lamada strain or other upcoming variant) with appalling infectivity and immune escape ability that may arise in the future. The missiles represent antibodies, and the houses represent host cells. The virus number of four variants in the cartoon is estimated according to the ratio of reproduction number (R0) of the variants to the original strain, and the number of viruses hit by missiles is estimated according to the immune escape ability [https://aci.health.nsw.gov.au/covid-19/critical-intelligence-unit/sars-cov-2-variants]. This cartoon depicts the characteristics including infectivity and immune escape ability of these VOCs. Infectivity: Delta> Alpha> Beta/ Gamma; immune escape ability: Beta/Gamma> Delta> Alpha. **b** Amino acid substitutions of four VOCs (Alpha, Beta, Gamma, and Delta variants) in spike protein that can cause immune escape. **c** Amino acid substitutions of four VOCs (Alpha, Beta, Gamma, and Delta variants) in spike protein that enhance the infectivity. **d** The neutralizations of four RBD-target antibodies (Bamlanivimab, Etesevimab, Casirivimab, and Imdevimab) (https://www.fda.gov) which have received emergency use authorization (EUA) for COVID-19 therapy, other four RBD-target antibodies (RBD-48, RBD-85, RBD-98, RBD-109) and four NTD-target antibodies (NTD-18, NTD-20, NTD-69, and NTD-71) isolated form convalescent sera against four VOCs and D614G mutant are displayed. The neutralizations against Alpha, Beta, Gamma, Delta variants, and D614G mutant are shown as red column, green column, blue column, purple column, and pink column respectively, the data are reproduced from the original article^1^ with permissions. **e** The neutralizing abilities of BNT162b2-immune sera and AZD1222-immune sera obtained at different times after vaccination against different variants. 4 means all participants maintained neutralization ability, 3 means 75% participants maintained neutralization ability, 2 means 50% participants maintained neutralization ability, 1 means 25% participants maintained neutralization ability, the data are reproduced from the original article^1^ with permissions

By 26 August 2021, COVID-19 has caused more than 214.6 million infections and 4,474,622 deaths (https://coronavirus.jhu.edu/). Vaccination is an effective means to prevent virus spreading and end the epidemic. WHO has authorized two inactivated vaccines (BBIBP-CorV, CoronaVac), two viral vector vaccines (AZD1222, Ad26.COV2-S) and two mRNA vaccines (mRNA1273, BNT162b2) to prevent SARS-CoV-2 infections (https://extranet.who.int/pqweb/vaccines/covid-19-vaccines). However, circulating variants including Delta have posed enormous challenges to the effectiveness of vaccines. Delta, also termed B.1.617.2 variant, detected in India in September 2020, had already spread to 115 countries (https://cov-lineages.org/global_report_B.1.617.2.html). Delta variant with a strong immune escape ability was considered as the most contagious variant known so far (Fig. 1a) (https://www.who.int/emergencies/diseases/novel-coronavirus-2019/media-resources/science-in-5/episode-45---delta-variant). Potential key sites causing immune escape (Fig. 1b) and enhancing viral infectivity (Fig. 1c) are summarized.

Planas D et al.^1^ found that larger syncytia appeared in S-fuse cells infected by Delta variant compared with Alpha variant and D614G mutant, indicating Delta variant may have higher cell fusion level and infectivity. Then, the three-dimensional structure of S protein was displayed, and several mutations of Delta variant including del156-157, G158R, L452R, T478K were speculated to result in the reduction of antibody neutralization. Subsequently, the neutralization efficiencies of eight receptor binding domain (RBD) antibodies (Bamlanivimab, Etesevimab, Casirivimab, Imdevimab, RBD-48, RBD-85, RBD-98, and RBD-109) and four N terminal domain (NTD) antibodies (NTD-18, NTD-20, NTD-69, and NTD-71) were evaluated. Among them, bamlanivimab completely lost its neutralization against Beta and Delta variants (Fig. 1d), and significantly neutralization activity reductions were found in one RBD antibody (RBD-85) and three NTD antibodies (NTD-20, NTD-69, NTD-71). It is worth noting that one dose of BNT162b2 vaccination after natural infection could provide effective protection against variant Alpha, Beta, and Delta. In addition, the authors tested the efficacy of neutralizing antibodies in vaccine sera from 16 BNT162b2 vaccine recipients and 23 AZD1222 vaccine recipients. None of the serum samples, collected three weeks after 1st dose of BNT162b2, can effectively neutralize Alpha, Beta and Delta variants, the similar result was also observed in the serum samples collected from 10 weeks after 1st dose of AZD1222. After 2nd dose of BNT162b2 for 5 weeks or 2nd dose of AZD1222 for 4 weeks, 94 and 95% sera effectively neutralized Delta variant, respectively, while 81 and 95% sera neutralized Beta variant. And the authors believed that 2nd dose of vaccine is critical to boost antibodies production, which is consistent with another recent research.^3^ Astoundingly, for the serum samples collected 13 weeks after 2nd dose of BNT162b2, only 46 and 85% sera retained the neutralization against Beta and Delta variants respectively (Fig. 1e), and the sharp decline of neutralization antibody level to Beta variant during 8 weeks indicates that long-term protection of vaccination needs to be monitored.

In addition, Peiyong Shi’s group evaluated the neutralizing efficiency of BNT162b2 vaccine sera on Kappa(B.1.617.1), Delta(B.1.617.2), B.1.617.v2, B.1.618, and Eta(B.1.525) variants.^2^ Based on the SARS-CoV-2 reverse genetic system developed previously, following variants were obtained using SARS-CoV-2 USA-WA1/2020 strain: (1) B.1.525-spike; (2) B.1.617.1-spike; (3) B.1.617.2-spike; (4) B.1.617.2-v2-spike and (5) B.1.618-spike. 20 vaccine-immune serum samples were collected for PRNT50 assay, the neutralization geometric mean titers (GMTs) ratios of the sera against above variants to USA-WA1/2020 virus were 0.64, 0.31, 0.70, 0.68, and 0.66, respectively. In addition, the RNA/PFU ratio was used to measure the infectivity of variants. Compared with other variants, Kappa and Delta have higher infectivity, which is consistent with the results of Planas D et al.^1^ Although Delta variant has higher infectivity and can cause immune escape, Liu et al believed that BNT162b2 is still effective for it. A recent study showed that Lambda variant (C.37) harboring L452Q, F490S has infected more than 80% population in Peru, suggesting it may have startling infectivity and immune escape ability.^4^ Fortunately, a new vaccine strategy using nanoparticles covered with antigens from multiple variants to manufacture vaccines with multi-immunogenicity can induce high-level and diverse antibody responses, it brings new hope for SARS-CoV-2 prevention and control in the future.^5^

In summary, these recent studies evaluated the transmission and immune escape abilities of the major circulating SARS-CoV-2 variant Delta, and emphasize the importance of continuous molecular surveillance. Meanwhile, these studies are significant for the guidance of the epidemic control and effective vaccination: (1) vaccination should be actively carried out regardless of whether infected by SARS-CoV-2 previously, and 2nd dose vaccination should be taken on time; (2) relevant information about vaccine seed strain should be updated in time according to the variants with higher infectivity and immune escape abilities; (3) new vaccination strategies containing multiple variant antigens in one vaccination should be considered and priorly scheduled; (4) variants with higher transmission and immune escape abilities are likely to appear in the very soon future, scientific and effective epidemic prevention measures including vaccination, wearing masks, washing hands frequently, maintaining social distance and avoiding crowd gathering should be strictly observed.
